# Efficiency score from data envelopment analysis can predict the future onset of hypertension and dyslipidemia: A cohort study

**DOI:** 10.1038/s41598-019-52898-9

**Published:** 2019-11-08

**Authors:** Sho Nakamura, Hiroto Narimatsu, Yoshinori Nakata, Masahiko Sakaguchi, Tsuneo Konta, Masafumi Watanabe, Yoshiyuki Ueno, Kenichi Ishizawa, Hidetoshi Yamashita, Takamasa Kayama, Takashi Yoshioka

**Affiliations:** 1 Department of Clinical Oncology, Yamagata University Faculty of Medicine, 2-2-2 Iida-nishi, Yamagata, Yamagata, 990-9585 Japan; 2 School of Health of Innovation, Kanagawa University of Human Services, 3-25-10 Research Gate Building 2-A, Tonomachi, Kawasaki-ku, Kawasaki, Kanagawa 210-0821 Japan; 3 Cancer Prevention and Control Division, Kanagawa Cancer Center Research Institute, 2-3-2 Nakao, Asahi-ku, Yokohama, Kanagawa 241-8515 Japan; 4 Department of Healthcare Management, Teikyo University Graduate School of Public Health, 2-11-1 Kaga, Itabashi-ku, Tokyo, 173-8605 Japan; 5 Deparment of Public Health, Yamagata University Graduate School of Medicine, 2-2-2 Iida-nishi, Yamagata, Yamagata, 990-9585 Japan; 6 Department of Cardiology, Pulmonology, and Nephrology, Yamagata University Faculty of Medicine, 2-2-2 Iida-nishi, Yamagata, Yamagata, 990-9585 Japan; 7 Department of Gastroenterology, Yamagata University Faculty of Medicine, 2-2-2 Iida-nishi, Yamagata, Yamagata, 990-9585 Japan; 8 Department of Neurology, Hematology, Metabolism, Endocrinology and Diabetology, Yamagata University Faculty of Medicine, 2-2-2 Iida-nishi, Yamagata, Yamagata, 990-9585 Japan; 9 Department of Ophthalmology and Visual Sciences, Yamagata University Faculty of Medicine, 2-2-2 Iida-nishi, Yamagata, Yamagata, 990-9585 Japan; 10 Global Center of Excellence, Yamagata University Faculty of Medicine, 2-2-2 Iida-nishi, Yamagata, Yamagata, 990-9585 Japan

**Keywords:** Epidemiology, Preventive medicine

## Abstract

Primary prevention focuses on ensuring that healthy people remain healthy. As it is practically difficult to provide intervention for an entire healthy population, it is essential to identify and target the *at risk of risks* population. We aimed to distinguish *at risk of risks* population using data envelopment analysis (DEA). Efficiency score was calculated from the DEA using a cohort sample and its association with the onset of hypertension and dyslipidemia was analyzed. A stratification analysis was performed according to the number of conventional risk factors in participants. The adjusted odds ratios (aORs) of the incidence of hypertension and dyslipidemia according to a 0.1-point increase in efficiency score were 0.66 (90% confidence interval [CI] 0.55–0.78, p < 0.0001) and 0.84 (90% CI 0.75–0.94, p = 0.01), respectively. In the stratification analysis, aOR of the incidence of hypertension according to a 0.1-point increase in efficiency score was 0.57 (90% CI 0.37–0.89, p = 0.04) in participants with no conventional risk factors. Participants with lower efficiency score were suggested to be at high risk for future onset of hypertension and dyslipidemia. The DEA might enable us to identify the risk of hypertension where conventional methods might fail.

## Introduction

Lifestyle diseases, such as hypertension, dyslipidemia, and obesity can be prevented to some extent by adhering to a healthy lifestyle. The high-risk approach and the population approach are two interventional approaches that aim to prevent lifestyle diseases. The former involves providing interventions for individuals with risk factors, while the latter provides an opportunity to increase the health level of the entire population^1^. For example, the specific health checkups conducted in Japan are one of the high-risk approach interventions, which focus on metabolic syndrome, whereby individuals whose body mass index (BMI) or waist circumference exceed the cut-offs are targeted^2,3^. This approach, however, cannot prevent healthy individuals from being at high risk for lifestyle diseases; the population-approach covers such healthy individuals. However, providing high-quality intervention to an entire population is impractical due to limited medical resources, and even if we could, the population-approach might increase the health inequality, which would hinder us from providing interventions for those who really need them^4^.

**Figure 1:**
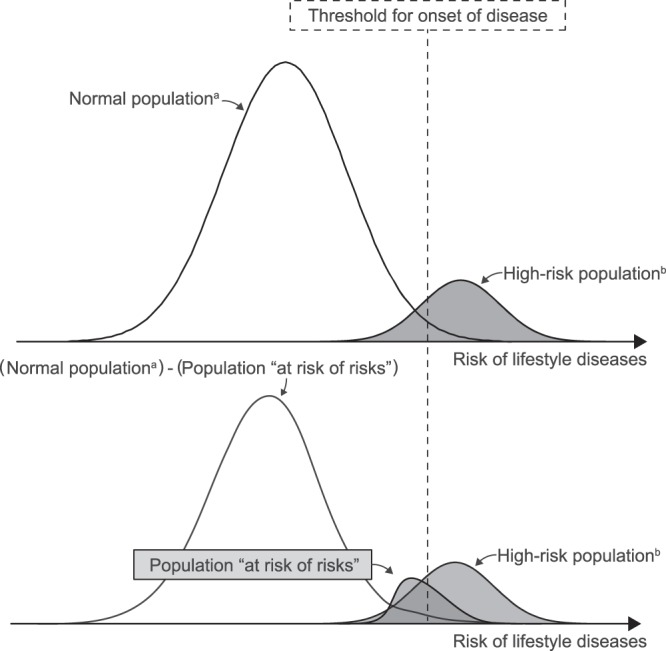
Conceptual diagram of population at risk of lifestyle diseases. The *at risk of risks* population refers to those who are at risk of a certain disease, although the results of their health-checkup are within the normal range. ^a^Population whose results of the health-checkup was within the normal range. ^b^Population identified as high-risk based on the results of the health-checkup.

To prevent healthy individuals from being at high risk for lifestyle diseases, the population *at risk of risks* should be identified (Fig. 1). Targeting primary preventive interventions in the *at risk of risks* population would make disease prevention more efficient. One possible strategy to distinguish the *at risk of risks* population is to use genomic information for risk stratification; however, such methods that can be applied in clinical practice to prevent lifestyle disease in the general population are not yet available. Another strategy, known as the vulnerable population approach, uses socio-economic status to identify the *at risk of risks* population^4^. However, there is no established indicator of socio-economic status that has generally been accepted.

Data envelopment analysis (DEA) is a method in operations research, mainly in business engineering and economics, to measure productive efficiency in a decision-making unit (DMU), such as business entities^5–7^. The greater the output to the input (higher output/input value) and the greater the profit to the required cost, the more efficient the DMU is^8^. Given that DMU is an individual factor and efficiency is a constitutional factor, efficiency could be considered as a risk factor for the disease of interest for each individual (DMU). We hypothesized that the DEA could be applied to identify the *at risk of risks* population for a certain lifestyle disease.

We previously tested this hypothesis and reported the findings with regard to evaluating the risk of obesity^9^. Considering each individual as a DMU, lifestyle practices (physical activity and energy intake) as input, and BMI as output, each individual’s *efficiency score* for BMI according to their lifestyle was calculated by the DEA. We observed an increased risk of obesity (higher BMI) in less efficient individuals. The DEA allowed us to evaluate the risk of obesity, without using unobserved confounders such as genetic information and socioeconomic status. Therefore, we sought to apply this method to other lifestyle diseases, such as hypertension and dyslipidemia.

In this study, we performed the DEA for healthy individuals and calculated their efficiency scores for blood pressure and serum cholesterol, as the risk factors for hypertension and dyslipidemia, respectively. We analyzed the association between the efficiency score and onset of hypertension and dyslipidemia using the data from a population-based prospective cohort study. Assuming that DEA can distinguish the *at risk of risks* population for hypertension and dyslipidemia, we might be able to provide more effective preventive interventions, which would ensure that healthy individuals remain healthy for longer, without being at risk for diseases.

## Methods

### Study population

We used data from the Yamagata Study (Takahata), a population-based prospective cohort study. The study design has been detailed elsewhere^10^. In brief, this cohort study was based on a health checkup, and data on the results of the checkup, such as anthropometric traits and laboratory data from the blood sample, were obtained. The baseline survey was conducted from 2004 to 2006. The follow-up survey was conducted from 2011 to 2012, 5–8 years after the baseline survey. This study was approved by the Ethics Committee of the Yamagata University Faculty of Medicine.

### Assessment of lifestyles and diseases

Nutritional intake was assessed using the brief self-administered diet history questionnaire, and information on daily intake of salt in grams, potassium in milligrams, and total energy in kilocalories (kcal) were also obtained^11^. Additionally, salt intake was estimated by Tanaka’s formula using urinary sodium and urinary creatinine; these were evaluated from the urine collected at the baseline survey and were used for sensitivity analysis^12^. Physical activity was assessed using the Japan Arteriosclerosis Longitudinal Study Physical Activity Questionnaire, by which the total energy and activity-specific energy can be quantified as metabolic equivalents-hours per day (METs-h/day)^13^.

Participants who had hypertension or dyslipidemia at baseline, or who had previously been diagnosed with either disease and were receiving treatment were excluded from the analysis. Undiagnosed participants meeting the following criteria at baseline were excluded from the analysis: presence of hypertension defined as systolic blood pressure ≥18.67 kPa (140 mmHg) or diastolic blood pressure ≥12.00 kPa (90 mmHg); presence of dyslipidemia defined as triglyceride (TG) ≥1.69 mmol/L (150 mg/dL), low-density lipoprotein cholesterol (LDL-C) ≥3.63 mmol/L (140 mg/dL), or high-density lipoprotein cholesterol (HDL-C) <1.04 mmol/L (40 mg/dL). The onset of hypertension or dyslipidemia was determined by the questionnaire or meeting the above diagnostic criteria at the follow-up survey.

### DEA analysis

We used the input-oriented constant returns-to-scale Charnes-Cooper-Rhodes model of DEA, given its ability to include multiple inputs and outputs without requiring an a priori function specification^14^. In this context, a DMU was defined as an entity, which is responsible for converting the inputs into outputs^15^; therefore, we defined the DMUs as each individual. In the model for hypertension, the inputs were the inverse of salt intake, the inverse of total energy intake, and physical activity, and the outputs were the inverse of systolic and diastolic blood pressure. In the model for dyslipidemia, the inputs were physical activity and the inverse of total energy intake, and the outputs were HDL-C, the inverse of TG, and the inverse of LDL-C. The inverse values were used to fit them into the definition of efficiency in DEA; efficiency is high when input is minimized while the outputs are held constant, or when the output is maximized while the inputs are held constant^16^. Each participant’s efficiency score was calculated using DEA-Solver-Pro Software (Saitech, Inc., Tokyo, Japan)^6^. Higher scores indicated higher efficiency^17^.

### Assessment of conventional risk

We also calculated risk based on the conventional method; we adopted the method used in specific health checkups^2^. This method was modified because waist circumference was not measured until the start of the specific health checkups in 2008, which was conducted following the baseline survey. The definitions of the conventional risk factors in this study are shown in Table 1. We counted the number of factors that fulfilled these criteria for each participant. As participants with a pre-existing disease were excluded, factor 3 and factor 9 were excluded for assessing conventional risk for hypertension and dyslipidemia, respectively.

**Table 1:** Conventional risk assessment factors.

Factor 1	Systolic blood pressure ≥17.33 kPa (130 mmHg)
Factor 2	Diastolic blood pressure ≥11.33 kPa (85 mmHg)
Factor 3	Receiving treatment for hypertension
Factor 4	Fasting blood glucose ≥5.55 mmol/l (100 mg/dl)
Factor 5	HbA_1c_ ≥0.06 (5.6%)
Factor 6	Receiving treatment for diabetes
Factor 7	Triglyceride ≥1.69 mmol/l (150 mg/dl)
Factor 8	High density lipoprotein cholesterol <1.04 mmol/l (40 mg/dl)
Factor 9	Receiving treatment for dyslipidemia
Factor 10	BMI ≥25 kg/m^2^
Factor 11	History of smoking

HbA_1c_, hemoglobin A_1c_; BMI, body mass index.

### Statistical analysis

We performed a logistic regression analysis to calculate the odds ratio (OR) of the incidence of hypertension and dyslipidemia. Four univariate models were analyzed, and explanatory variables were efficiency scores in 2 models and conventional risk factors in 2 models. We adjusted the models with the efficiency scores by conventional risk factors, baseline age, sex, and baseline BMI. As a sensitivity analysis for the models in hypertension, we also included daily potassium intake as an adjustment factor. Further, we performed a stratification analysis according to the 4 groups stratified by the number of conventional risk factors; participants with no conventional risk factors (low-risk), those with one risk factor (moderate-risk), those with two risk factors (high-risk), and those with three or more risk factors (extreme-risk). In the logistic regression analysis, we assessed whether the continuous variables were linear on the logit using a generalized additive model with a smoothing spline using the gam function of the mgcv package in R^18^ and a Box-Tidwell test^19^. Any variable that could not achieve linearity on the logit as a continuous variable was categorized into that model (Supplementary Figs S1, S2). Age was categorized into ten-year groups (40–49, 50–59, 60–69, over 70 years), and body mass index (BMI) was categorized into two groups (<23, ≥23 kg/m2). Multicollinearity was assessed using the variance inflation factor (VIF) with the vif function of the DAAG package^20^, and receiver operating characteristic (ROC) curves after multivariate logistic regression models were illustrated using the roc function of the pROC package in R^21^. Statistical analyses were performed using R software (version 3.4.1)^22^.

## Results

### Baseline characteristics

**Figure 2:**
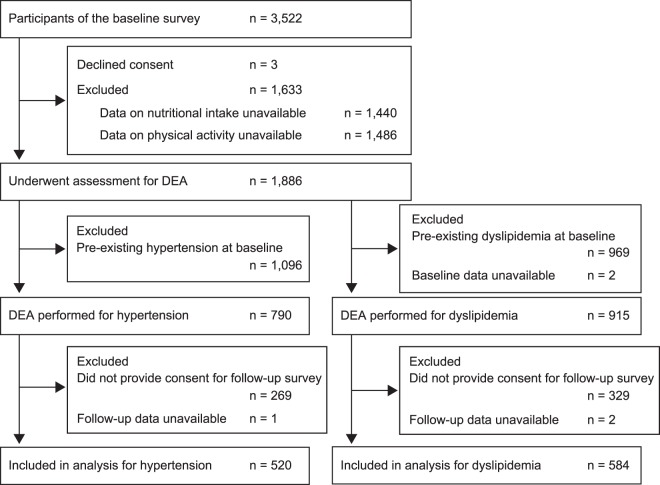
Flow diagram of the study participants. DEA, data envelopment analysis.

As shown in Fig. 2, of the 3522 participants of the Yamagata Study (Takahata), the efficiency scores by DEA were calculated for 790 participants for hypertension and 915 for hypertension and dyslipidemia. Data on the incidence of hypertension was available from 520 participants, and incidence of dyslipidemia was available from 584 participants.

Participant characteristics are shown in Table 2. The mean follow-up time of the participants was 5.6 (standard deviation [SD] 1.2) and 5.7 (SD 1.2) years, for hypertension and dyslipidemia, respectively. Hypertension and dyslipidemia was observed in 173/520 (33.3%) and 207/584 (35.4%) participants, respectively. The efficiency score of hypertension ranged from 0.45 to 1.0 (Mean 0.68; SD 0.11), and of dyslipidemia ranged from 0.33 to 1.0 (Mean 0.59; SD 0.13). Details of the results from the DEA (lambda values and assessment of excess use) and sensitivity analysis between the two estimating methods for salt intake are described in the Supplementary Information (Supplementary Text, Supplementary Figs. S3, S4, and Tables S1–S4).

**Table 2:** Participant characteristics.

	Dataset for hypertension	Dataset for dyslipidemia
*Characteristics*	n = 520	n = 584
Efficiency score	0.68 (0.11)	0.59 (0.13)
Conventional risk score		
0	141 (27.1%)	157 (26.9%)
1	150 (28.8%)	169 (28.9%)
2	110 (21.2%)	153 (26.2%)
3	91 (17.5%)	92 (15.8%)
4	26 (5.0%)	13 (2.2%)
5	2 (0.4%)	0 (0.0%)
*Baseline characteristics*		
Age (years)	57.2 (8.8)	60.2 (9.6)
40–49	100 (19.2%)	89 (15.2%)
50–59	212 (40.8%)	163 (27.9%)
60–69	160 (30.8%)	228 (39.0%)
70–	48 (9.2%)	104 (17.8%)
Sex (Women)	296 (56.9%)	300 (51.4%)
BMI (kg/m^2^)	22.6 (2.8)	22.9 (3.0)
≥23	221 (42.5%)	264 (45.2%)
Systolic blood pressure (mmHg)	121.6 (11.4)	131.4 (16.2)
Diastolic blood pressure (mmHg)	73.8 (8.5)	78.0 (10.7)
Triglycerides (mg/dL)	97.7 (67.2)	78.7 (26.7)
Low density lipoprotein cholesterol (mg/dL)	126.2 (29.0)	110.7 (19.0)
High density lipoprotein cholesterol (mg/dL)	60.3 (14.2)	63.3 (13.7)
Energy intake (kcal/day)	2214.3 (617.7)	2291.1 (638.8)
Salt intake (g/day)	12.4 (3.5)	12.9 (3.8)
Potassium intake (g/day)	2.6 (1.0)	2.7 (1.1)
Physical activity (METs-hr/day)	37.3 (5.8)	36.7 (5.7)
*Characteristics at follow-up*		
Follow-up duration (years)	5.6 (1.2)	5.7 (1.2)
BMI (kg/m^2^)	22.6 (3.0)	22.9 (3.1)
Systolic blood pressure (mmHg)	127.5 (15.8)	134.3 (18.6)
Diastolic blood pressure (mmHg)	76.6 (9.6)	78.1 (10.5)
Triglycerides (mg/dL)	108.3 (67.6)	91.9 (50.6)
Low density lipoprotein cholesterol (mg/dL)	123.7 (29.0)	111.7 (25.0)
High density lipoprotein cholesterol (mg/dL)	60.1 (14.4)	62.5 (14.7)
Incidence of the disease of interest	173 (33.3%)	207 (35.4%)

Data are shown in mean (standard deviation) unless otherwise specified. BMI, body mass index; METs, metabolic equivalents; hr, hour.

### Logistic regression analysis

The ORs of the incidence of hypertension and dyslipidemia are shown in Table 3. The adjusted ORs of the incidence of hypertension and dyslipidemia according to a 0.1-point increase in efficiency score were 0.66 (90% confidence interval [CI] 0.55–0.78, p < 0.0001) and 0.84 (90% CI 0.75–0.94, p = 0.01), respectively. The evidence for association between the conventional risk and onset of the diseases appeared to be weak compared to that of the efficiency score, especially in the low-risk group of hypertension (OR 1.42 [90% CI 0.87–2.34, p = 0.24]). Results of the sensitivity analysis of adjusting the model with potassium intake are shown in the Supplementary Information (Supplementary Text and Supplementary Table S5).

**Table 3:** Odds ratios for the onset of hypertension and dyslipidemia

	Crude odds ratio (90% CI)	P Value	Adjusted odds ratio (90% CI)^a^	P Value
*Hypertension*				
Efficiency score (0.1 point)	0.64 (0.55–0.75)	<0.0001	0.66 (0.55–0.78)	<0.0001
Risk level^b^				
Low risk	Reference		Reference	
Moderate risk	1.44 (0.95–2.18)	0.15	1.42 (0.87–2.34)	0.24
High risk	3.13 (2.08–4.70)	<0.0001	1.85 (1.11–3.10)	0.049
Extreme risk	4.40 (2.95–6.58)	<0.0001	2.18 (1.26–3.77)	0.02
*Dyslipidemia*				
Efficiency score (0.1 point)	0.84 (0.75–0.94)	0.01	0.84 (0.75–0.94)	0.01
Risk level^b^				
Low risk	Reference		Reference	
Moderate risk	2.10 (1.47–2.98)	0.001	1.87 (1.23–2.84)	0.01
High risk	1.84 (1.28–2.64)	0.01	1.74 (1.12–2.73)	0.04
Extreme risk	2.05 (1.39–3.04)	0.003	1.82 (1.08–3.04)	0.06

CI, confidence interval. Odds ratio calculated using logistic regression analysis. Efficiency score calculated using data envelopment analysis.

^a^Variables in the models are efficiency score, conventional risk score, age, sex, body mass index at baseline.

^b^Participants with none, one, two, and three or more of the conventional risk factors were classified as low-, moderate-, high-, and extreme-risk groups, respectively.

### Stratification analysis

The results of the stratification analysis are shown in Table 4. In the models for hypertension, the efficiency score was highest in the low-risk group (0.72 [SD 0.10]) and lowest in extreme-risk group of participants having three or more conventional risk factors (0.65 [SD 0.10]). A higher efficiency score was associated with a decreased risk of hypertension; adjusted ORs of the incidence of hypertension according to a 0.1-point increase in efficiency score were 0.57 (90% CI 0.37–0.89, p = 0.04), 0.65 (90% CI 0.48–0.88, p = 0.02), 0.50 (90% CI 0.34–0.72, p = 0.002), and 0.82 (90% CI 0.59–1.13, p = 0.32) in low-, moderate-, high-, and extreme-risk groups, respectively. Evidence for the association between the efficiency score and dyslipidemia was weak; adjusted ORs of the incidence of dyslipidemia according to a 0.1-point increase in efficiency score were 0.79 (90% CI 0.60–1.05, p = 0.18), 0.80 (90% CI 0.65–0.99, p = 0.08), 0.93 (90% CI 0.77–1.13, p = 0.53), and 0.86 (95% CI 0.63–1.17, p = 0.41) in low-, moderate-, high-, and extreme-risk groups, respectively.

**Table 4:** Results of the logistic regression analysis stratified by conventional risk.

	Subgroups according to the number of conventional risk factors^a^							
	Low-risk group		Moderate-risk group		High-risk group		Extreme-risk group	
*Hypertension*	n = 141		n = 150		n = 110		n = 119	
Efficiency score (SD)	0.72 (0.10)		0.70 (0.12)		0.66 (0.11)		0.65 (0.10)	
Incidence	26 (18.4%)		41 (27.3%)		45 (40.9%)		61 (51.3%)	
LR analysis	Odds ratio (90% CI)	P value	Odds ratio (90% CI)	P value	Odds ratio (90% CI)	P value	Odds ratio (90% CI)	P value
Unadjusted	0.58 (0.39–0.89)	0.03	0.75 (0.57–0.996)	0.10	0.61 (0.44–0.85)	0.01	0.89 (0.66–1.20)	0.52
Adjusted^b^	0.57 (0.37–0.89)	0.04	0.65 (0.48–0.88)	0.02	0.50 (0.34–0.72)	0.002	0.82 (0.59–1.13)	0.32
*Dyslipidemia*								
	n = 157		n = 169		n = 153		n = 105	
Efficiency score (SD)	0.60 (0.12)		0.59 (0.13)		0.62 (0.15)		0.55 (0.13)	
Incidence	40 (25.5%)		69 (40.8%)		56 (36.6%)		42 (40.0%)	
LR analysis	Odds ratio (90% CI)	P value	Odds ratio (90% CI)	P value	Odds ratio (90% CI)	P value	Odds ratio (90% CI)	P value
Unadjusted	0.78 (0.59–1.02)	0.13	0.81 (0.65–0.99)	0.09	0.90 (0.75–1.08)	0.34	0.85 (0.65–1.11)	0.31
Adjusted^b^	0.79 (0.60–1.05)	0.18	0.80 (0.65–0.99)	0.08	0.93 (0.77–1.13)	0.53	0.86 (0.63–1.17)	0.41

SD, standard deviation; LR, logistic regression; CI, confidence interval. Odds ratio calculated using logistic regression analysis. Efficiency score calculated using data envelopment analysis.

^a^Participants with none, one, two, and three or more of the conventional risk factors were classified as low-, moderate-, high-, and extreme-risk groups, respectively.

^b^Variables in the models are efficiency score, conventional risk score, age, sex, and body mass index at baseline.

## Discussion

In this study, we found that the higher the efficiency scores calculated by the DEA, the lower the risk of hypertension and dyslipidemia. This suggests that efficiency scores could be useful for assessing the risk of both diseases. The efficiency scores were higher in the low-risk group than in the higher risk groups, which is consistent with the defined concept of performing DEA to evaluate the risk; the results of the DEA showed equivalent validity to the conventional method in assessing the risk of hypertension and dyslipidemia.

The results of the stratification analysis indicated the potential of the DEA to distinguish the *at risk of risks* population for hypertension. An increase in the efficiency score was associated with a decrease in the risk for hypertension in the low- to high-risk groups, whereas, the efficiency score could not predict the onset of hypertension in the extreme-risk group. In other words, the efficiency score calculated by the DEA could not distinguish the inequality in the level of risk among the participants in the extreme-risk group, who had three or more than three conventional risk factors. Accumulations of conventional risk factors seemed to surpass the risks that could be distinguished by the efficiency score. However, participants in the extreme-risk group can be regarded as *at risk* rather than *at risk of risks*, and it is more likely for them to be identified by the conventional secondary-prevention method heretofore, such as the specific health check-ups carried out in Japan^2,3^. On the other hand, the conventional method is inadequate to precisely classify the *at risk of risks* population in low- to high-risk groups, especially in the low-risk, thereby making it difficult to provide adequate intervention owing to insufficient resources (manpower). Taken together, the efficiency score calculated by the DEA may serve as a risk stratification measure to classify the *at risk of risks* population, thus enabling us to provide primary preventive intervention.

Contrary to the findings observed for hypertension, we did not observe a clinically meaningful association between the risk of dyslipidemia and the efficiency score calculated by the DEA in the subgroups stratified by the number of conventional risk factors. Moreover, the relationship between the risk of these diseases and conventional risk factors appeared to be different from that observed for hypertension. The ORs for the onset of hypertension were higher in the higher risk groups. On the other hand, the ORs for the onset of dyslipidemia appeared to be equivalent in the three groups stratified by the conventional risk factors compared to low-risk group (Table 3). Having one conventional risk factor increased the risk of the onset of dyslipidemia; however, additional risk factors did not further increase the risk. One explanation for this finding is that dyslipidemia is a spectrum of diseases with various subtypes according to the type of lipoproteins. Further, some conventional risk factors, such as diabetes, obesity, and smoking, are known to affect the level of blood cholesterol; part of which is referred to as secondary dyslipidemia. We assumed that clinically irrelevant findings observed in the stratification analysis for dyslipidemia were due to the imprecise stratification of the risk for dyslipidemia by conventional risk factors. Nevertheless, we cannot currently provide an explanation that goes beyond our speculation regarding those discrepancies in observed results between hypertension and dyslipidemia; future research should explore this topic further.

Our results suggest that the efficiency score from the DEA and conventional risk factors could be used in a mutually-complementary manner in an actual healthcare setting. Combining both strategies for risk stratification would enable us to provide the primary prevention more efficiently, as we would be able to distinguish the *at risk of risks* population. In the domain of business administration, where DEA originates, efficiency score is used as a benchmark to improve the management of each DMU by targeting the efficiency frontier (those whose efficiency score is 1). However, our premise for applying the findings of this study is to use efficiency score as a cross-sectional risk to relatively evaluate inequality in the risk for hypertension and dyslipidemia. In this way, the efficiency score could encompass the effect of unobserved factors, such as genetic and socio-economic factors, lean body mass, renal function and so on. A a more specific example is a genetic variation rs8022678, a single nucleotide variant that has been suggested as affecting the sodium sensitivity of an individual (further discussion can be found in Supplementary Text)^23^. This difference is caused by the discrepancy between the efficient state and healthy state of the individual. For example, an efficient individual is someone who leads a sedentary lifestyle and consumes excessive energy (calories) and salt, although their blood pressure is under control. On the other hand, even if their blood pressure is well-controlled, leading a healthy lifestyle is still ideal because there are many other diseases that a healthy lifestyle could prevent. Being efficient does not indicate being excluded from the recommended healthy lifestyle habits. The efficiency score and conventional risk factors can be used together to determine whether interventions are needed. As an example for hypertension, those at an extreme risk according to the conventional risk factors require intervention regardless of their efficiency score, but for individuals at low- to high-risk, who are often deemed normal, we can provide intervention with priority to those with a low efficiency score. With respect to dyslipidemia, we can put priority in those with moderate- to extreme-risk. Combining the efficiency score from the DEA and conventional risk factor might enable us to identify the *at risk of risk* population before they become *at risk*, especially for hypertension.

We previously investigated the association between efficiency score from the DEA and obesity^9^; the results showed no association between the efficiency score and change in BMI (difference in the BMI in the follow-up period). The following reasons might explain the inconsistent finding; first, the prevalence of obesity is known to reach its peak at 50–60 years of age, and the majority of the study participants were around this age range^24^. Thus, the average BMI converged to zero, and only a slight change in the BMI was observed for most participants. Contrary to our previous findings, in this study, we observed the onset of the disease in more than 30% of the participants. This might have contributed to our findings that efficiency score could predict the onset of hypertension and dyslipidemia. Second, the gene-environment interaction is known to affect the onset of obesity^25^. Input used for the DEA regarding obesity might have been affected by the gene-environment interaction, to a certain extent; thus, the efficiency score from the DEA could become insufficient for identifying the inequalities in the risk levels. However, the efficiency score calculated for the risk evaluation of hypertension was useful even for the population with no conventional risk factors in the stratified analysis. This indicates that the application of the efficiency score might be more efficient for the primary prevention of hypertension, in a population with no conventional risk factor. There are several other limitations that need to be acknowledged. We could not assess the change in the efficiency score; the efficiency score might change according to aging or because of some of the factors assumed to be encompassed by the DEA as mentioned above. In addition, because the detailed questionnaires on the lifestyle were only obtained during the baseline survey, we could not assess the alteration in the participants’ lifestyles during the follow-up period. Although we used a validated method to assess nutritional intake and physical activity^11,13^, self-reported nature of our study hindered us from avoiding bias caused by measurement error. Furthermore, while our results demonstrate the potential of DEA to be applied to the domain of healthcare, there are only limited available data to support our findings in the field of primary preventive medicine. To determine the practicality of the efficiency score from the DEA for primary preventive intervention, we need to demonstrate that targeting the *at risk of risks* population segregated by efficiency score can prevent the disease onset. We aim to conduct an interventional study from 2019, based on the hypothesis that the *at risk of risks* population can be identified using the efficiency score from the DEA.

## Conclusions

Efficiency score calculated by the DEA could be used to identify those *at risk of risks* for a particular disease among healthy individuals at baseline. Notably, the efficiency score distinguished the inequality in the risk of hypertension in the low-risk group. Efficiency score has the potential to be applied to primary preventive intervention.

## Supplementary information

Supplementary Information

## Data Availability

The data analyzed during the current study are not publicly available for ethical reasons. The data that support the findings of this study can be made available after approval for data access by application to Yamagata University.
