# Supplementary material for: Efficiency score from data envelopment analysis can predict the future onset of hypertension and dyslipidemia: A cohort study
Source: Sci Rep. 2019 Nov 8;9:16309. doi: 10.1038/s41598-019-52898-9 (PMC6841950; doi:10.1038/s41598-019-52898-9)
Supplement: Supplementary file 1 — Supplementary Information [file 41598_2019_52898_MOESM1_ESM.pdf]

Supplementary information for "Efficiency score from data envelopment analysis can predict the future onset of hypertension and dyslipidemia: A cohort study."

Sho Nakamura, Hiroto Narimatsu, Yoshinori Nakata, Masahiko Sakaguchi, Tsuneo Konta, Masafumi Watanabe, Yoshiyuki Ueno, Kenichi Ishizawa, Hidetoshi Yamashita, Takamasa Kayama, Takashi Yoshioka

Table of contents

		Pages
1.	Supplementary Text	2–3
2.	Supplementary Figures	4–7
3.	Supplementary Tables	8–12

Supplementary Text

RESULTS

Data envelopment analysis

We performed DEA for hypertension using two estimating methods for salt intake; salt intake was obtained from a brief self-administered diet history questionnaire, and estimated salt intake was calculated from urine sodium and urine creatinine at baseline. Comparison of the two efficiency scores are shown in Supplementary Figure S3.

Results of the data envelopment analysis are shown in Supplementary Tables S1 and S2, for hypertension and dyslipidemia, respectively. There were 12 efficient (efficiency score = 1) participants (A to L DMU) in hypertension, and 10 (O to X DMU) in dyslipidemia. We showed the least efficient 5 DMUs in Additional Tables 1 and 2; M to Q DMU in hypertension and Y to AC DMU in dyslipidemia. Among these DMUs, we selected 4 least efficient DMUs, that is P, Q, AB, and AC DMU, and calculated excess use in these DMUs using lambda values (weight of the peers) shown in Supplementary Tables S1 and S2. Lambda values show the variables related to the constraints limiting the efficiency of each unit to be no greater than 1. These results are shown in Supplementary Tables S3 and S4; for example, although Q DMU had less salt intake by 8.5 g, less energy intake by 1814.7 kcal, and more physical activity equivalent to 26.0 METs-h/day, he was at a risk of hypertension (inefficient) due to his predisposition.

Logistic regression analysis

In the logistic regression analysis, BMI was categorized into the unstratified model for dyslipidemia. The linearity of age on the logit for the incidence of dyslipidemia in participants with three or more conventional risk factor seemed to be non-linear; however, using categorized age in this model inflated the VIF value to 10.5. Hence, we used uncategorized age. The largest VIF value among all the models was 2.29, indicating that there was no collinearity in the models. Interaction terms were not added to the model for a reliable analysis, because none of the interaction terms were significant in the model, and they inflated the VIF value to an unacceptable range. Results of the two sensitivity analyses that were performed are shown in Supplementary Table S5; 1) efficiency score using estimated salt intake from urinary analysis, and 2) adjusting the model of hypertension with potassium intake.

ROC curves of the efficiency scores for the onset of hypertension and dyslipidemia after the multivariate logistic regression analysis are shown in Supplementary Figure S4.

DISCUSSION

Results of the sensitivity analysis showed comparable results with the primary models shown in the main manuscript.

As described above and in Supplementary Tables S3 and S4, inefficient DMUs had better lifestyles than do the efficient DMUs; however, their health status—blood pressure and serum cholesterol levels—were poorer than that of the efficient DMUs. Factors such as genetic, socio-economic, lean body mass, and renal function for example, could be affecting this difference in the efficiency; we assume that the combined effect of these and other confounding factors, including unknown ones, is equivalent to the excess use observed in each DMU. As an example, Q DMU is predisposed to the risk of hypertension that equals the intake of 8.5-g salt, taking 1814.7-kcal energy, and being sedentary as equivalent to 26.0 METs-h/day.

Supplementary Figures

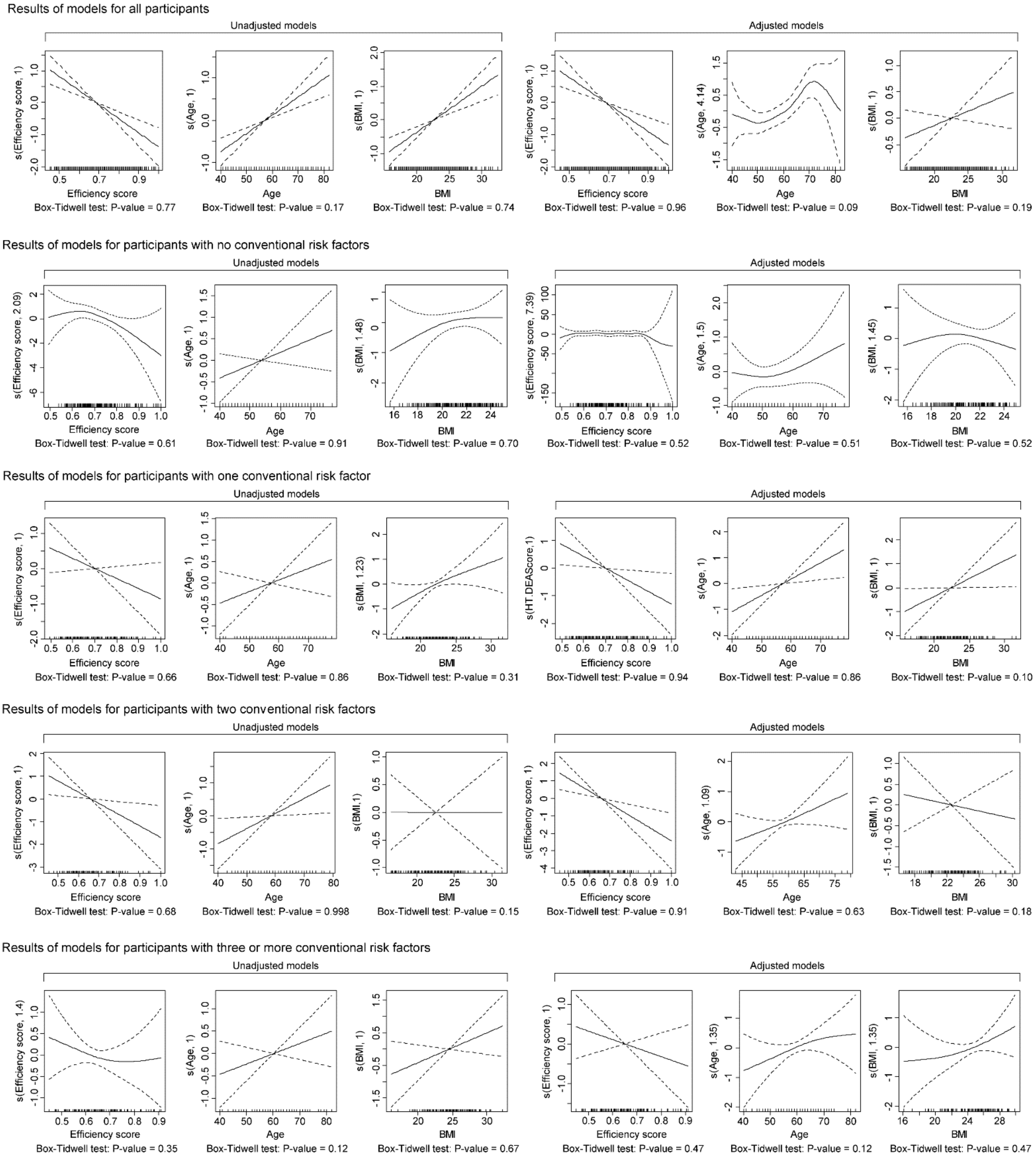

Supplementary Figure S1. Assessment of linearity assumption of each logistic regression model for hypertension using smoothing spline and Box-Tidwell test. BMI, body mass index.

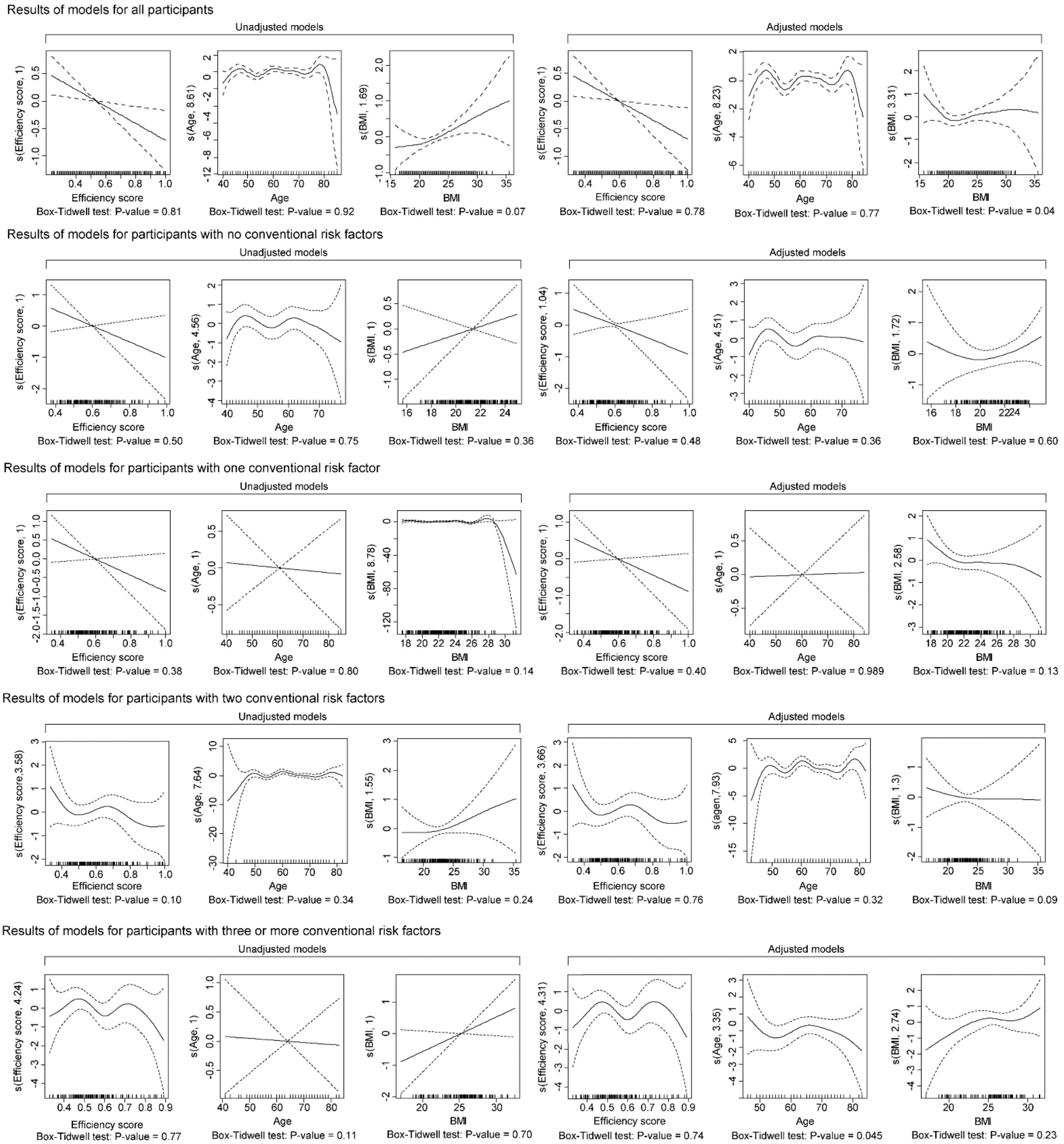

Supplementary Figure S2. Assessment of linearity assumption of each logistic regression model for dyslipidemia using smoothing spline and Box-Tidwell test. BMI, body mass index.

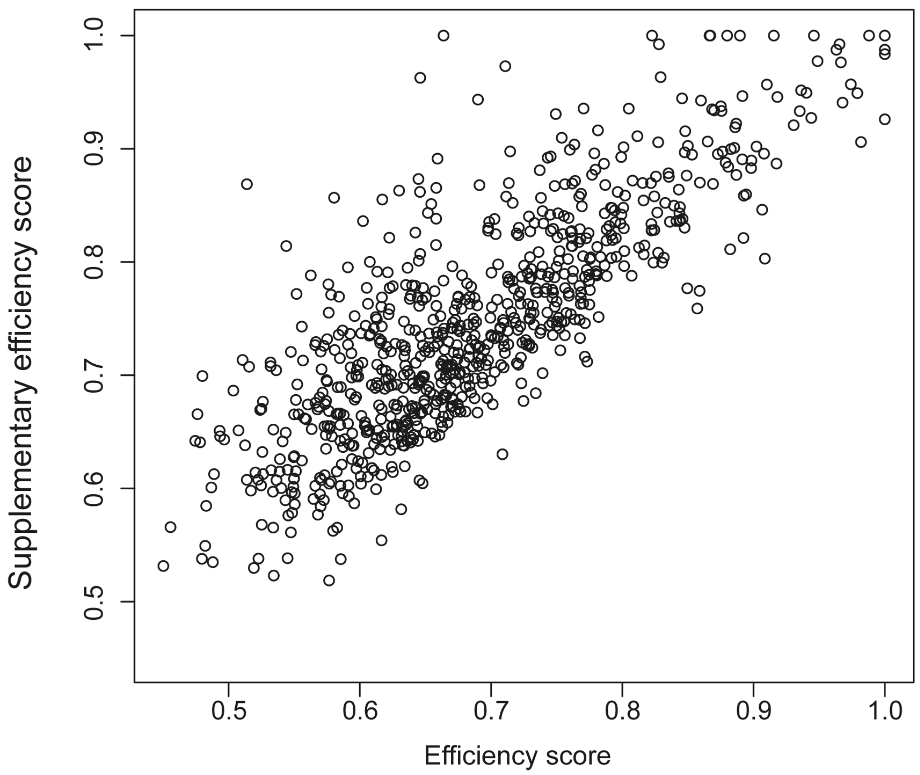

Supplementary Figure S3. Comparison of the two efficiency scores for hypertension. The salt intake used as the input for efficiency score on the x-axis was acquired from the brief self-administered diet history questionnaire, and the supplementary efficiency score on the y-axis was estimated from urine sodium and urine creatinine.

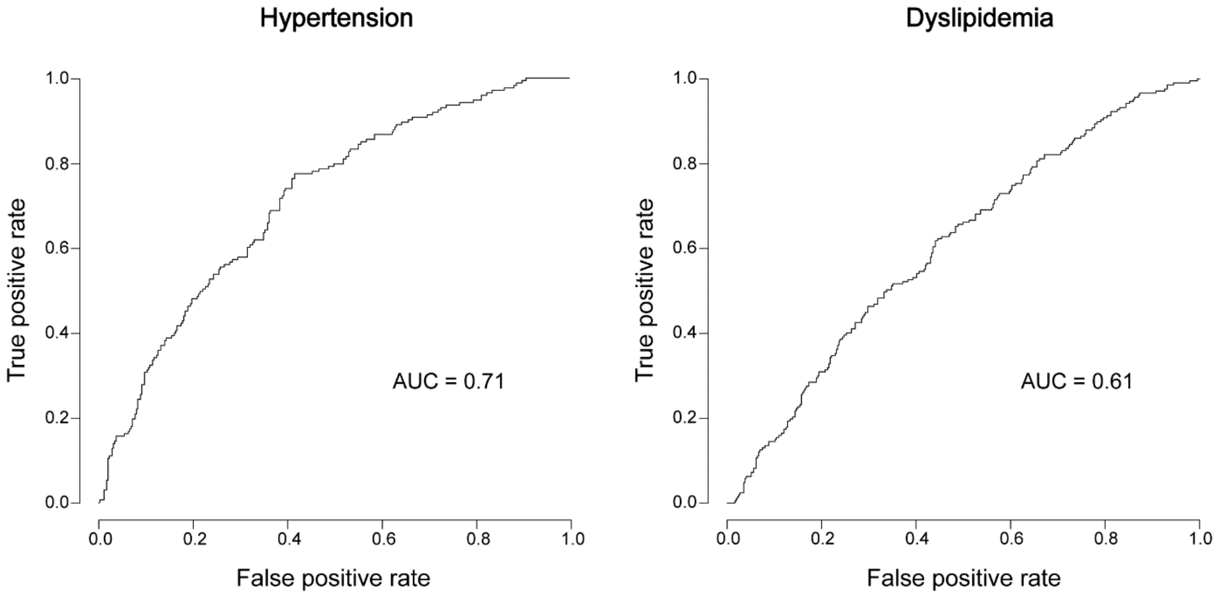

Supplementary Figure S4. Receiver operating characteristic curves of the efficiency score. AUC, area under the curve.

Supplementary Table S1. Details of decision making units in hypertension

DMU	Efficiency score	Salt intake (g/day)	Energy intake (kcal/day)	Physical activity (METs-hr/day)	Blood Pressure (mmHg)		Reference set (peers)	Weights of the peers (lambdas)												Peer count
					Systolic	Dyastolic		A	B	C	D	E	F	G	H	I	J	K	L	
A	1.000	17.4	4181.7	29.3	118	68.0	A	1.000	0.000	0.000	0.000	0.000	0.000	0.000	0.000	0.000	0.000	0.000	0.000	83
B	1.000	21.5	2924.2	31.6	100	62.0	B	0.000	1.000	0.000	0.000	0.000	0.000	0.000	0.000	0.000	0.000	0.000	0.000	265
C	1.000	23.5	4783.3	30.0	112	80.0	C	0.000	0.000	1.000	0.000	0.000	0.000	0.000	0.000	0.000	0.000	0.000	0.000	176
D	1.000	19.6	2721.6	34.2	98	50.0	D	0.000	0.000	0.000	1.000	0.000	0.000	0.000	0.000	0.000	0.000	0.000	0.000	237
E	1.000	18.8	2770.3	38.1	100	50.0	E	0.000	0.000	0.000	0.000	1.000	0.000	0.000	0.000	0.000	0.000	0.000	0.000	26
F	1.000	10.1	1650.8	31.3	102	50.0	F	0.000	0.000	0.000	0.000	0.000	1.000	0.000	0.000	0.000	0.000	0.000	0.000	22
G	1.000	10.3	2524.5	30.0	98	60.0	G	0.000	0.000	0.000	0.000	0.000	0.000	1.000	0.000	0.000	0.000	0.000	0.000	330
H	1.000	10.7	1683.1	31.5	90	52.0	H	0.000	0.000	0.000	0.000	0.000	0.000	0.000	1.000	0.000	0.000	0.000	0.000	260
I	1.000	10.5	1753.6	32.1	92	50.0	I	0.000	0.000	0.000	0.000	0.000	0.000	0.000	0.000	1.000	0.000	0.000	0.000	77
J	1.000	11.9	2154.2	42.6	96	40.0	J	0.000	0.000	0.000	0.000	0.000	0.000	0.000	0.000	0.000	1.000	0.000	0.000	6
K	1.000	7.1	851.7	27.6	94	62.0	K	0.000	0.000	0.000	0.000	0.000	0.000	0.000	0.000	0.000	0.000	1.000	0.000	43
L	1.000	23.1	3154.6	43.5	100	60.0	L	0.000	0.000	0.000	0.000	0.000	0.000	0.000	0.000	0.000	0.000	0.000	1.000	24
Five participants with lower efficienct score in low-risk group																				
M	0.578	15.6	1661.9	42.6	128.0	82.0	B, H	0.000	0.760	0.000	0.000	0.000	0.000	0.000	0.019	0.000	0.000	0.000	0.000	0
N	0.573	12.4	2693.5	47.6	126.0	78.0	A, C, D, E	0.476	0.000	0.370	0.013	0.047	0.000	0.000	0.000	0.000	0.000	0.000	0.000	0
O	0.569	10.8	1873.3	49.8	112.0	68.0	B, C, D, G	0.000	0.392	0.148	0.153	0.000	0.000	0.208	0.000	0.000	0.000	0.000	0.000	0
P	0.568	6.5	1143.2	42.8	116.0	70.0	H, K	0.000	0.000	0.000	0.000	0.000	0.000	0.000	0.719	0.000	0.000	0.059	0.000	0
Q	0.512	8.9	1904.4	53.4	120.0	70.0	A, C, D, G	0.365	0.000	0.120	0.183	0.000	0.000	0.225	0.000	0.000	0.000	0.000	0.000	0

DMU, decision making unit; METs-hr, metabolic equivalents-hour.

Supplementary Table S2. Details of decision making units in dyslipidemia

DMU	Efficiency score	Energy intake (kcal/day)	Physical activity (METs-h/day)	Triglycerides (mg/dL)	HDL cholesterol (mg/dL)	LDL cholesterol (mg/dL)	Reference set (peers)	Weights of the peers (lambdas)										Peer count
								O	P	Q	R	S	T	U	V	W	X	
O	1.000	847.3	32.7	80	60	22	O	1.000	0.000	0.000	0.000	0.000	0.000	0.000	0.000	0.000	0.000	89
P	1.000	3636.5	41.6	45	81	68	P	0.000	1.000	0.000	0.000	0.000	0.000	0.000	0.000	0.000	0.000	101
Q	1.000	3072.8	33.0	27	102	81	Q	0.000	0.000	1.000	0.000	0.000	0.000	0.000	0.000	0.000	0.000	405
R	1.000	1550.7	33.4	49	60	26	R	0.000	0.000	0.000	1.000	0.000	0.000	0.000	0.000	0.000	0.000	192
S	1.000	3100.5	35.6	80	108	105	S	0.000	0.000	0.000	0.000	1.000	0.000	0.000	0.000	0.000	0.000	43
T	1.000	4127.2	40.1	61	97	119	T	0.000	0.000	0.000	0.000	0.000	1.000	0.000	0.000	0.000	0.000	33
U	1.000	3674.7	28.1	122	67	73	U	0.000	0.000	0.000	0.000	0.000	0.000	1.000	0.000	0.000	0.000	109
V	1.000	2524.5	30.0	55	101	110	V	0.000	0.000	0.000	0.000	0.000	0.000	0.000	1.000	0.000	0.000	193
W	1.000	4962.1	34.3	40	67	99	W	0.000	0.000	0.000	0.000	0.000	0.000	0.000	0.000	1.000	0.000	53
X	1.000	2609.5	28.5	41	94	66	X	0.000	0.000	0.000	0.000	0.000	0.000	0.000	0.000	0.000	1.000	328
Five participants with lower efficienct score in low-risk group																		
Y	0.424	1578.7	38.1	71	53	132	Q, X	0.000	0.000	0.031	0.000	0.000	0.000	0.000	0.000	0.000	0.530	0
Z	0.410	1661.9	42.6	74	58	131	V, X	0.000	0.000	0.000	0.000	0.000	0.000	0.000	0.191	0.000	0.411	0
AA	0.395	1873.3	49.8	83	55	107	Q, R, U	0.000	0.000	0.398	0.057	0.000	0.000	0.164	0.000	0.000	0.000	0
AB	0.391	2154.2	42.6	93	44	117	Q, R, U	0.000	0.000	0.235	0.059	0.000	0.000	0.245	0.000	0.000	0.000	0
AC	0.391	1354.0	50.6	64	61	81	O, R, X	0.012	0.000	0.000	0.073	0.000	0.000	0.000	0.000	0.000	0.595	0

DMU, decision making unit; METs-hr, metabolic equivalents-hour; HDL, high density lipoprotein; LDL, Low density lipoprotein.

Supplementary Table S3. Example of estimating the effect of the unobserved factors in inefficient decision-making units in hypertension.

	Peer 1[a]			Peer 2[a]			Peer 3[a]			Peer 4[a]			Sum of weighted values (inversed value)	Values of the DMU	Excess use by DMU
	Values (inversed value)	Weight of the peer (lambda)	Weighted value	Values (inversed value)	Weight of the peer (lambda)	Weighted value	Values (inversed value)	Weight of the peer (lambda)	Weighted value	Values (inversed value)	Weight of the peer (lambda)	Weighted value			
Q DMU (efficiency score = 0.512)															
Salt intake (g/day)	17.4 (0.0576)	0.3654	0.0210	23.5 (0.0426)	0.1203	0.0051	19.6 (0.0509)	0.1828	0.0093	10.3 (0.0971)	0.2251	0.0219	0.0573 (17.4)	8.9	-8.5
Energy intake (kcal/day)	4181.7 (0.0002)	0.3654	0.0001	4783.3 (0.0002)	0.1203	0.00003	2721.6 (0.0004)	0.1828	0.0001	2524.5 (0.0004)	0.2251	0.0001	0.0003 (3719.1)	1904.4	-1814.7
Physical activity (METs-hr/day)	29.3	0.3654	10.7	30.0	0.1203	3.6	34.2	0.1828	6.3	30.0	0.2251	6.7	27.3	53.4	26.0
Blood pressure (mmHg)	118/68			112/80			98/50			98/60				120/70	
P DMU (efficiency score = 0.568)															
Salt intake (g/day)	10.7 (0.0937)	0.7193	0.0674	7.1 (0.1401)	0.0590	0.0083							0.0757 (13.2)	6.5	-6.8
Energy intake (kcal/day)	1683.1 (0.0006)	0.7193	0.0004	851.7 (0.0012)	0.0590	0.0001							0.0005 (2013.3)	1143.2	-870.0
Physical activity (METs-hr/day)	31.5	0.7193	22.7	27.6	0.0590	1.6							24.3	42.8	18.5
Blood pressure (mmHg)	90/52			94/62										116/70	

DMU, decision making unit; METs-hr, metabolic equivalents-hour.

[a]Peers for M DMU are A , C, D, and G for peer 1 to 4 respectively. Peers for N DMU are H, and K for peer 1, and 2 respectively.

Supplementary Table S4. Example of estimating the effect of the unobserved factors in inefficient decision-making units in dyslipidemia.

	Peer 1[a]			Peer 2[a]			Peer 3[a]			Sum of weighted values (inversed value)	Values of the DMU	Excess use by DMU
	Values (inversed value)	Weight of the peer (lambda)	Weighted value	Values (inversed value)	Weight of the peer (lambda)	Weighted value	Values (inversed value)	Weight of the peer (lambda)	Weighted value			
AC DMU (efficiency score = 0.391)												
Energy intake (kcal/day)	847.3 (0.0012)	0.0117	0.00001	1550.7 (0.0006)	0.0728	0.00005	2609.5 (0.0004)	0.5950	0.0002	3463.2 (0.0003)	1354.0	-2109.154
Physical activity (METs-h/day)	32.7	0.0117	0.3832	33.4	0.0728	2.4330	28.5	0.5950	16.9796	19.8	50.6	30.8
Triglycerides (mg/dL)	80			49			41.0				64	
HDL cholesterol (mg/dL)	60			60			94				61	
LDL cholesterol (mg/dL)	22			26			66				81	
AB DMU (efficiency score = 0.391)												
Energy intake (kcal/day)	3072.8 (0.0003)	0.2353	0.0001	1550.7 (0.0006)	0.0593	0.00004	3674.7 (0.0003)	0.2455	0.0001	5506.9 (0.0002)	2154.2	-3352.7
Physical activity (METs-h/day)	33.03911	0.2353	7.7726	33.4	0.0593	1.9823	28.1	0.2455	6.8930	16.6	42.6	25.9
Triglycerides (mg/dL)	27			49.0			122				93	
HDL cholesterol (mg/dL)	102			60.0			67				44	
LDL cholesterol (mg/dL)	81			26			73				117	

DMU, decision making unit; METs-hr, metabolic equivalents-hour; HDL, high density lipoprotein; LDL, Low density lipoprotein.

[a]Peers for AC DMU are O, R, and X for peer 1 to 3 respectively. Peers for AB DMU are Q, R, and U for peer 1 to 3 respectively.

Supplementary Table S5. Results of the sensitivity analysis for the models of hypertension

	Crude odds ratio (90% CI)	P Value	Adjusted odds ratio (90% CI)	P Value
Efficiency score using salt intake estimated from urinary sodium and creatinine				
Supplementary efficiency score (0.1 point)	0.65 (0.55-0.76)	< 0.0001	0.67 (0.56-0.80)[a]	0.0002
Model adjusted with potassium intake				
Efficiency score (0.1 point)	-	-	0.59 (0.48-0.71)[b]	< 0.0001

CI, confidence interval.

Odds ratio calculated using logistic regression analysis. Efficiency score calculated using data envelopment analysis.

[a] Variables in the models are efficiency score, conventional risk score, age, sex, and body mass index at baseline.

[b] Variables in the models are efficiency score, conventional risk score, age, sex, body mass index, and potassium intake at baseline.
